# Surgical Correction of Simple Cutaneous Syndactyly of the Third and Fourth Fingers in a Three-Year-Old Girl: A Case Report

**DOI:** 10.7759/cureus.91455

**Published:** 2025-09-02

**Authors:** Stanislaw Szymkiewicz

**Affiliations:** 1 Department of Urology, Janusz Korczak Provincial Specialist Hospital in Słupsk, Słupsk, POL

**Keywords:** case report, congenital anomaly, pediatric hand surgery, syndactyly, z-plasty

## Abstract

Syndactyly is a common congenital malformation of the hand, characterized by fusion of adjacent digits. Early surgical correction is recommended to prevent functional limitations and esthetic concerns. We report the case of a three-year-old girl with congenital simple complete cutaneous syndactyly between the third and fourth fingers of the left hand. The fusion was limited to soft tissues, with fully formed and separate fingernails. Surgical separation was performed using interdigital dorsal and palmar Z-plasty incisions, followed by full-thickness skin grafting harvested from the groin donor site. Postoperative recovery was uneventful. At follow-up, the patient demonstrated a normal range of motion, satisfactory esthetic results, and no evidence of web creep or joint stiffness. This case supports the effectiveness and safety of Z-plasty combined with full-thickness skin grafting for the correction of simple syndactyly in early childhood.

## Introduction

Syndactyly is one of the most common congenital anomalies of the upper extremities, with an incidence of approximately 1 in 2,000 live births [1]. Syndactyly is classified as simple (cutaneous), complex (involving bone or nails), or complicated (associated with other anomalies). Fusion may be complete (to the fingertip) or incomplete. While it often occurs as an isolated anomaly, syndactyly can also be syndromic, including patterns such as acrosyndactyly, cleft hand, or synpolydactyly [2].

Early surgical intervention is generally recommended to prevent functional impairment and to achieve acceptable cosmetic results [1,3]. If left untreated, syndactyly may lead to progressive functional limitation, joint contractures, and cosmetic deformity due to differential finger growth [1]. Among available methods, interdigital Z-plasty is preferred to avoid longitudinal scar lines and contracture, while full-thickness skin grafting is commonly selected in complete webs to maintain web depth when local tissue is insufficient [3,4].

## Case presentation

**Figure 1:**
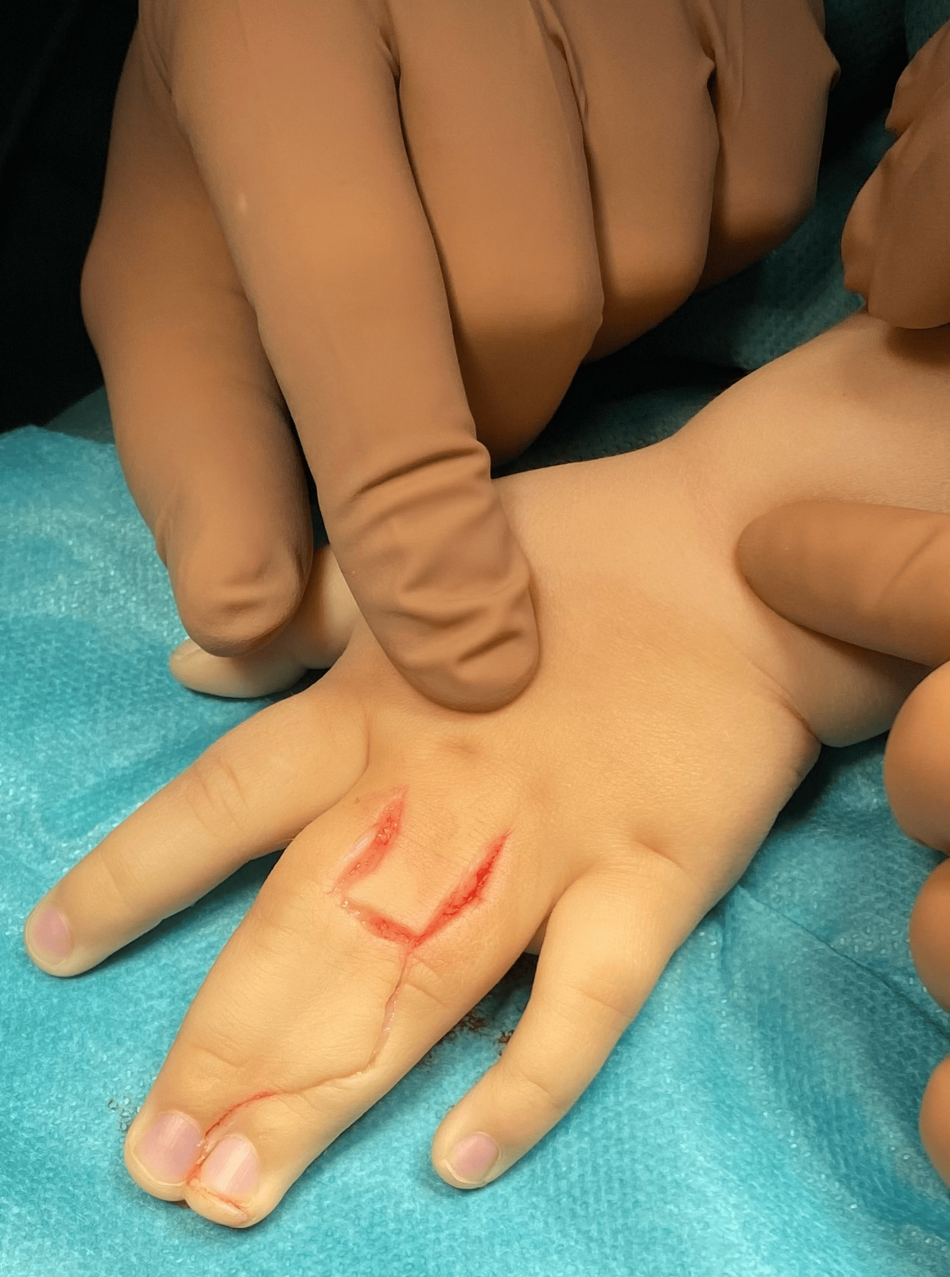
Preoperative markings for syndactyly separation (dorsal aspect) with Z-plasty design for web reconstruction

**Figure 2:**
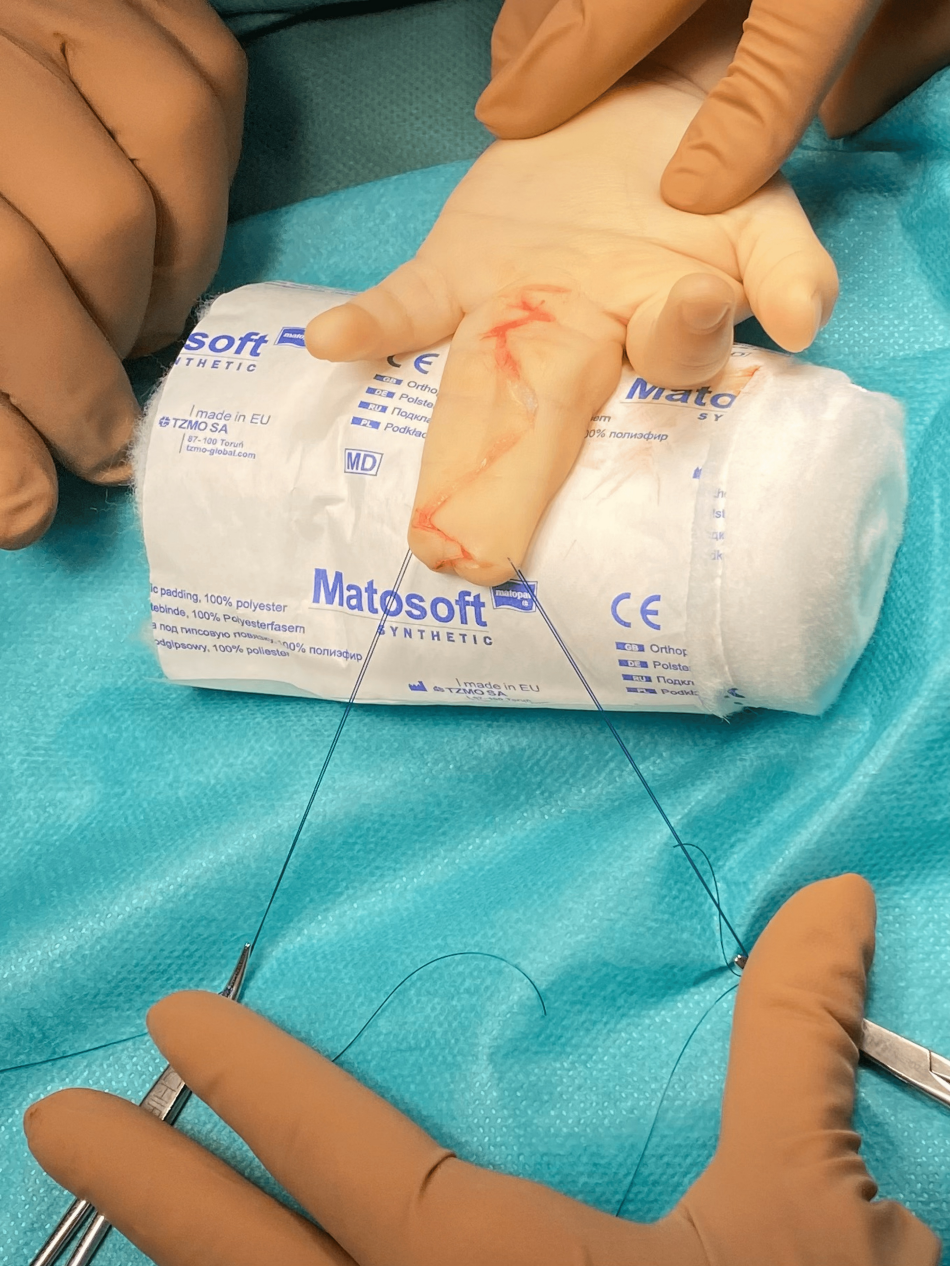
Initial incision and positioning (palmar aspect) before interdigital dissection

**Figure 3:**
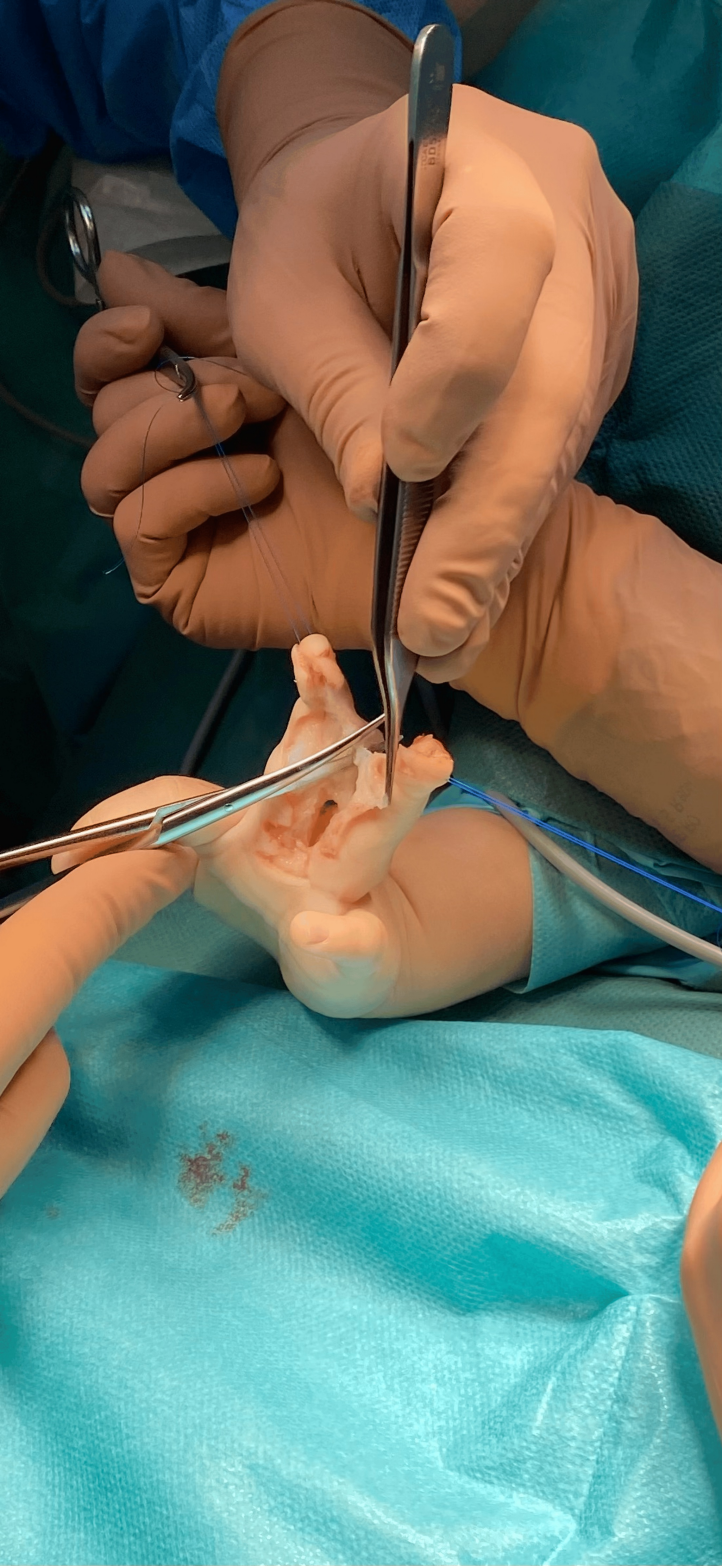
Intraoperative dissection of the cutaneous bridge with preservation of digital neurovascular bundles

**Figure 4:**
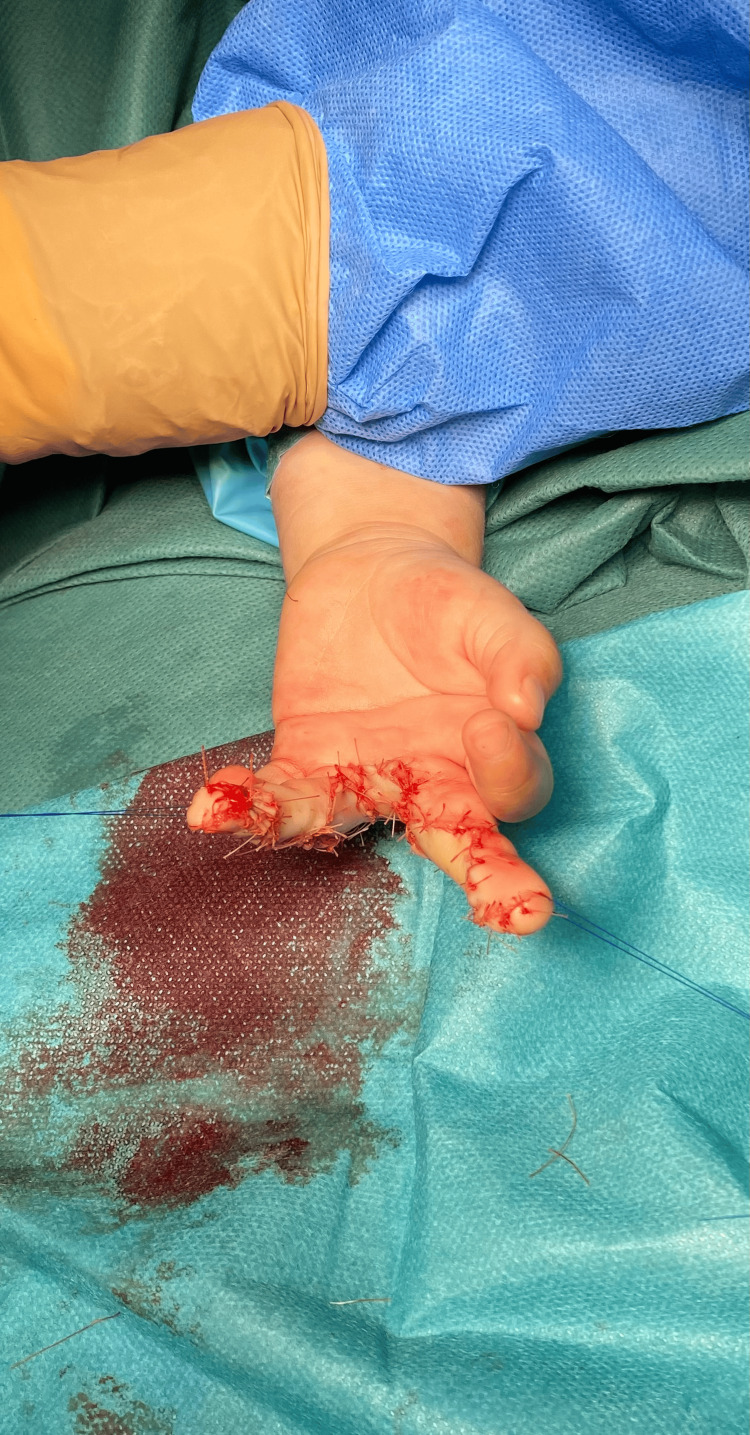
Immediate postoperative view (palmar aspect) after closure and web reconstruction

A three-year-old, right-handed girl was referred to the pediatric surgery department due to congenital fusion of the third and fourth digits of the left hand. The fusion had been noted at birth and had remained unchanged since then. The child was otherwise healthy, with no significant medical history or associated congenital anomalies. Family history was negative for congenital hand deformities, and there was no consanguinity between the parents. Physical examination revealed soft tissue syndactyly between the third and fourth fingers, classified as simple complete cutaneous syndactyly, extending from the base to the tip of the fingers. The fingernails were separate and fully formed, with no signs of bony fusion. The hand displayed a good range of passive motion, but the webbing restricted finger abduction, impairing grasp and pinch. Clinical examination confirmed a normal range of passive motion at the metacarpophalangeal (MCP) and interphalangeal (IP) joints. No additional congenital anomalies or syndromic features were identified [2]. Radiographs were not obtained because separate, fully formed nails, normal joint mobility, and absence of deformity reliably excluded bony fusion on clinical grounds; imaging is reserved for suspected osseous involvement [3]. The child’s growth and psychomotor development were within normal limits. After discussion with the parents, elective surgical correction was scheduled. The procedure was performed under general anesthesia with a regional nerve block for postoperative pain control. An interdigital dorsal and palmar Z-plasty incision pattern was designed to reconstruct the web space while avoiding linear scarring and minimizing contracture formation [3]. After meticulous dissection of the cutaneous bridge and preservation of neurovascular structures, the fingers were fully separated. The newly created interdigital web space was covered with full-thickness skin grafts (FTSGs) harvested from the groin donor site. Hemostasis was carefully achieved, and the grafts were sutured in place with absorbable material. The donor site was closed primarily [3,4]. A soft padded dressing and protective dorsal splint were applied to immobilize the fingers in slight abduction. Intraoperative steps are shown in Figures 1-4.

The dressing was removed on the fifth postoperative day, and the graft showed good integration. The hand was immobilized using a dorsal splint in slight finger abduction for a total of three weeks. After splint removal, the patient began a supervised occupational therapy program focusing on range of motion, fine motor skills, and scar massage. Therapy sessions were conducted twice weekly for six weeks [3].

Although no standardized functional outcome scores were formally recorded, clinical assessment at the six-month follow-up demonstrated that the total active motion (TAM) of the affected fingers was within the normal age range. No web creep, hypertrophic scarring, or joint stiffness was observed [1,3].

## Discussion

Surgical correction of syndactyly is typically recommended between one and three years of age, as early intervention minimizes the risk of secondary deformities due to differential finger growth and joint contractures [1,3]. The present case, operated on at three years of age, aligns with these recommendations and achieved excellent short-term functional and cosmetic results [1,3].

Our approach utilized interdigital dorsal and palmar Z-plasty incisions combined with FTSGs from the groin, a strategy widely advocated in complete cutaneous webs to preserve web depth and reduce the risk of linear scar contracture when local tissue is insufficient [3,4]. The zigzag incision technique, popularized to avoid longitudinal scars and subsequent contracture formation, remains a preferred method for web space reconstruction in simple syndactyly [3]. In their systematic review and meta-analysis, Schirlo et al. compared techniques for congenital syndactyly and reported differences across selected outcomes, underscoring the need to tailor the method to the web type and available tissue rather than assume a universally superior approach [4]. Likewise, Braun et al. emphasize meticulous neurovascular preservation and tension-free graft placement as key determinants of functional recovery and sensory outcomes [3].

While some authors advocate dorsal rectangular flaps to minimize donor-site morbidity in selected cases, this approach may show less favorable cosmetic blending in the interdigital space for complete syndactyly; in contrast, distant FTSG provides durable coverage and maintains web depth when local advancement is limited [2-4]. Our findings also support the observation that early correction in simple cutaneous syndactyly without bony involvement typically results in a near-normal range of motion and low complication rates [1].

Contemporary evidence syntheses reaffirm that technique selection should be individualized. A recent systematic review in Cureus provides a broad overview of timing and operative strategies for syndactyly release across age groups and web patterns [5], while a meta-analysis in Hand compares outcomes across techniques and highlights trade-offs, with no single method consistently outperforming others across all endpoints [4]. In complete cutaneous webs with limited local tissue, Z-plasty with FTSG remains a pragmatic option to maintain web depth and minimize linear contracture risk [3,4].

Limitations and future directions

This report reflects a single patient with short-term follow-up, which limits generalizability. Comparative studies in larger cohorts are needed to evaluate the relative effectiveness of techniques across web types and age at surgery [4,5]. A longer follow-up will help assess the durability of function and cosmesis and detect late complications such as web creep, hypertrophic scarring, or the need for revision surgery. Objective scores (e.g., standardized functional and scar assessments) were not recorded at baseline and will be incorporated in future visits [3].

## Conclusions

Early correction of simple complete syndactyly using interdigital Z-plasty with full-thickness skin grafting can yield excellent short-term functional and cosmetic outcomes. These findings are consistent with contemporary literature and support an individualized, anatomy-driven choice of technique rather than a one-size-fits-all approach.
